# Development and validation of prediction models for special subtype of primary aldosteronism: patients with negative adrenal CT imaging

**DOI:** 10.3389/fendo.2025.1563748

**Published:** 2025-07-11

**Authors:** Hong Zhao, Pan Hu, Min Mao, Xin Li, Ling Wang, Jing Chang

**Affiliations:** ^1^ Department of Cardiovascular Medicine, Cardiovascular Research Center, The First Affiliated Hospital of Chongqing Medical University, Chongqing, China; ^2^ The First Affiliated Hospital of Chongqing Medical University, Chongqing, China

**Keywords:** machine learning, AI, radiomics, primary aldosteronism, subtype diagnosis

## Abstract

**Objective:**

Current subtype diagnosis of primary aldosteronism relies on adrenal venous sampling and imaging, each with inherent limitations. Lesional adrenal glands with negative CT Imaging is a distinct subtype of primary aldosteronism that has been less frequently studied. The aim of this study was to develop and validate a machine learning and AI model for distinguishing adrenals with transversely negative lesions from normal adrenals Primary Aldosteronism.

**Materials and methods:**

We conducted a single-center retrospective study, assessing transverse adrenal scans of 170 PA patients. A specialized iterative method was employed for radiomic feature selection. Subsequently, six conventional machine learning methodologies were utilized to construct the radiomics models. This original data was subsequently applied in the construction of a radiomic model, which was combined with clinical data for the final model construction.

**Results:**

107 radiomic features were extracted from the adrenal scans and 10 features were selected for ML and AI modeling. In the clinical data, values for serum potassium, aldosterone excretion, uric acid, and IVSd were utilized in the model construction. The integration of clinical data further enhanced the model’s performance, with an AUC reaching 0.868 in the derived cohort, and an AUC of 0.853 in the temporal validation cohort.

**Conclusion:**

The study indicates that clinical-radiomic scores can independently serve as diagnostic biomarkers for the specialized PA subtype categorization. We give the proposal for the precise categorization concept in establishing a clinical-radiomic model for PA subtype diagnosis. The model demonstrates substantial potential for both clinical and translational research.

## 1 Introduction

Primary aldosteronism (PA) is a condition characterized by unilateral or bilateral adrenal glands autonomously overproducing aldosterone (1). Compared to patients with primary hypertension, individuals with PA have a higher incidence of cardiovascular diseases and other complications (2, 3). PA is the leading cause of secondary hypertension, with an occurrence rate of approximately 5% among hypertensive patients, and between 17% and 23% in patients with resistant hypertension (4–8). The diagnosis of PA generally involves screening for blood pressure, serum potassium, serum aldosterone, renin, and the ratio of the latter two confirmatory tests, including the captopril suppression test, intravenous saline load test, oral high-sodium diet test, and fludrocortisone suppression test, are then performed. These confirmatory tests have a sensitivity and specificity that can reach upwards of 80% or even over 90%. Consequently, as clinicians’ understanding of this disease deepens, the detection rate of PA in clinical practice has been increasing, and the misdiagnosis rate has been gradually decreasing (9, 10). However, it is important to note that there is no complete international consensus on some diagnostic details, such as the conditions for blood sampling, the methods for detecting aldosterone and renin, as well as the methods and diagnostic thresholds for screening and confirmatory tests (11). These differences can also affect the precision of PA diagnosis.

A current clinical challenge lies in the subtyping of PA—specifically determining the side of the adrenal lesion and its nature. The subtype diagnosis of PA directly influences the selection of subsequent treatment options (medication or surgery) (7, 9, 12). PA can be divided into aldosterone-producing adenoma (APA), idiopathic hyperaldosteronism (IHA) and other unilateral hyperplasia lesions, etc. according to histopathological manifestations (13). Clinically, PA can also be categorized into PA with positive cross-sectional imaging and PA with negative cross-sectional imaging (14). Studies suggest that approximately 30% of PA cases are considered negative on cross-sectional imaging (15), and other research summarizes that about half of the patients with PA have clinically evident disease, but no detectable nodules in routine cross-sectional imaging (14). Currently, methods used for subtyping in clinical practice include imaging examinations (adrenal CT or MRI) (9), radionuclide PET/CT scans (16, 17), and adrenal venous sampling (AVS—the gold standard for subtyping) (7–9). Among imaging examinations, CT is relatively inexpensive but less accurate (18). It cannot identify minor changes (as mentioned, PA negative on cross-sectional imaging), nor can it differentiate between functional and non-functional lesions. The accuracy of MRI is not superior to CT (7). PET/CT is expensive and a novel means of subtyping; its accuracy requires further validation (17). Adrenal vein sampling has high accuracy, with both sensitivity and specificity reaching over 90%. It is the recognized gold standard for subtyping both in China and internationally, but it is the most expensive and involves invasive surgical procedures (7, 9). Authoritative research shows that inconsistencies between CT/MRI and AVS results can be as high as 30% or more, leading to the most severe consequences such as erroneous removal of normal adrenals or subjecting patients to unnecessary surgical treatments (19, 20). In addition, radionuclide examinations and AVS require high capability of medical staff and high-grade medical equipment, making them difficult to implement in county hospitals (3, 21). There are also some predictive models based on clinical data, such as serum potassium, serum aldosterone, and urinary aldosterone, which predict whether the patient has unilateral or bilateral adrenal lesions. However, these studies have certain drawbacks. They only use imaging manifestations that can reflect the characteristics of a single adrenal gland and lack other data that can reflect the features of a single adrenal gland. Therefore, they are helpless in distinguishing whether adrenals negative on cross-sectional imaging have lesions (3, 22).

We contemplated whether a simple, inexpensive, and highly accurate method of subtyping could be obtained, and this study aims to seek a solution for the aforementioned challenges. With the rise of radiomics in recent years, methods for distinguishing between benign and malignant tissues and predicting disease prognosis through radiomics have been widely applied (23). Regarding adrenal-related topics, there are also articles on distinguishing adrenal hyperplasia from lipid-poor adenomas (24), aldosterone-producing adenomas from cortisol-producing adenomas (25), functional adrenal adenomas from non-functional adenomas (26), and lipid-poor adenomas from non-adenomas (27). To our knowledge, although some researchers have developed radiomics models for predicting PA subtypes (28, 29), no studies have reported on using CT-based radiomics to determine whether adrenals negative on CT cross-sectional imaging in PA patients are the lesion-bearing adrenals. Therefore, in this study, we developed and validated a subtype diagnosis model that integrates RFs and clinical characteristics, paving the way for future development of artificial intelligence in medical imaging.

## 2 Method

### 2.1 Data collection

**Figure 1:**
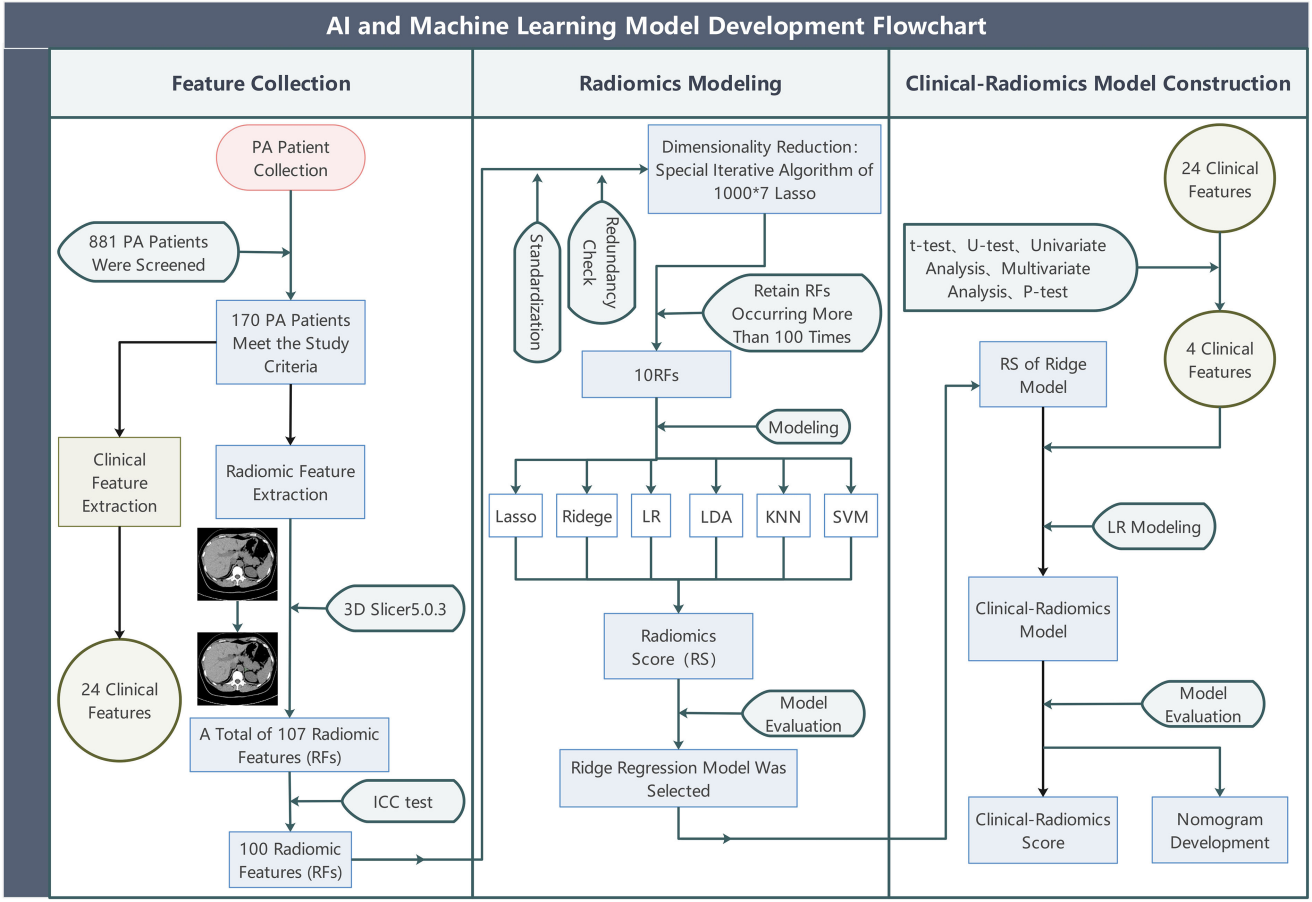
The overall study flow. PA, (primary aldosteronism); Lasso, (least absolute shrinkage and selection operator); LR, (Logistic Regression); Ridge, (Ridge Regression); LDA, (linear discriminant analysis); KNN, (k-nearest neighbors); SVM, (support vector machine).

The overall study design is shown in **Figure 1**. Data for all patient cases in this study were obtained from our hospital (single-center) from January 2012 to January 2024, screening 881 instances of confirmed PA accessible from our hospital. To develop and validate the predictive model, this study retrospectively recruited two independent cohorts (the derivation cohort and the temporal validation cohort), all of whom were patients diagnosed with PA at our hospital (diagnosed by at least one internationally recognized clinical diagnostic test). A total of 170 patients were ultimately included, comprising 120 individuals from the derivation cohort (49 males and 71 females) and 50 from the temporal validation cohort (24 females and 26 males), with their ages restricted to between 18 and 75 years old. We defined the meaning of positive and negative adrenal CT images; positive refers to the adrenal gland appearing enlarged, thickened, nodular, adenomatous, etc., on CT images, while negative refers to the adrenal gland being deemed morphologically normal by the radiologist upon reviewing the CT images. We used whether a patient’s single adrenal met the criteria as the main inclusion criterion. We defined the experimental group as patients with one adrenal gland appearing negative on CT images (cross-sectionally negative), but in a successful adrenal vein sampling, this side was confirmed to be the dominant secreting side. We labeled these as Label 1. Conversely, the control group consisted of patients with one adrenal gland appearing negative on CT images (cross-sectionally negative), and in a successful adrenal vein sampling, this side was confirmed to be the non-dominant secreting side. These adrenals did not exhibit the conditions of the experimental group, and we labeled these as Label 0. The experimental group and control group each had 85 members with available non-enhanced CT image data and clinical data. In the derivation cohort, CT images of the adrenal glands of 60 experimental group patients and 60 healthy control group patients were collected before December 31, 2022, and retrieved from the imaging system of our hospital. Participants in the temporal validation cohort, including 25 experimental group patients and 25 control group patients, had CT images collected after December 31, 2022. We excluded patients with the following conditions: ① patients with potential other adrenal diseases such as hypercortisolism, adrenal medullary lesions, adrenal metastases, etc.; ② those found to have lost or corrupted CT images in subsequent analyses; ③Poor imaging quality or unclear delineation between the adrenal gland and surrounding tissues in CT images made it indistinguishable even for resident doctors with two years of work experience and senior chief physicians.

We also retrospectively collected the following indicators: Age(years), Gender(male/female), Height(cm), Weight(kg), Body Mass Index (kg/m^2^), Systolic Blood Pressure(mmHg), Diastolic Blood Pressure(mmHg), Duration of Hypertension (months), Maximum Aldosterone (pg/ml), Renin (μIU/ml), Minimum Serum Potassium(mmol/L), Echocardiography-Interventricular Septal Thickness at End-Diastol (mm), Echocardiography-Left Ventricular Ejection Fraction (%), Renal Function-Uric Acid (μmol/L), Renal Function-Creatinine (μmol/L), Renal Function-Estimated Glomerular Filtration Rate (eGFR), Urine - Urinary Microalbumin/Creatinine Ratio (mg/g Cr), Urine-Potassium (mmol/L), Urine-24-hour Urinary Potassium Excretion (mmol/24h), Blood Lipids-Total Cholesterol (TC) (mmol/L), Blood Lipids-Triglycerides(TG) (mmol/L), Blood Lipids-Low-Density Lipoprotein Cholesterol (mmol/L), Blood Lipids-High-Density Lipoprotein Cholesterol (mmol/L), 24-hour Aldosterone Excretion (Maximum)(ng/24h). In the tables or figures of this article, they are abbreviated respectively as: Age, Gender, Height, Weight, BMI, SBP, DBP, HTN Duration, Aldo-max, Renin, K^+^(Min.), Echo-IVSd, Echo-LVEF, RF-UA, RF-Cr, RF-EGFR, U-MA/Cr, U-K 24h, U-K, BL-TC, BL-TG, BL-LDL, BL-HDL, 24h AE(Max.). The blood pressure value refers to the highest blood pressure during the patient’s disease course. The blood potassium refers to the lowest blood potassium during the patient’s disease course. Serum aldosterone refers to the highest random aldosterone index during the disease course. The 24-hour urinary aldosterone level refers to the highest value measured during the disease course. Test results from hospitals that conform to regional result recognition are equally recognized.

The data collection and analysis were approved by the Institutional Review Board of our hospital and followed the principles of the Helsinki Declaration. The requirement for informed consent was waived by our hospital’s Ethics Committee for this study. (Number: K2023-583).

### 2.2 Segmentation of ROI and extraction of RFs

All CT scans were obtained utilizing an identical multidetector CT system (Somatom Sensation 64; Siemens Healthineers, Erlangen, Germany) in accordance with a standardized protocol. The procedure involved scanning each patient (the study required only the non-enhanced phase of the scan). The parameters for the CT scan included a tube voltage of 100 kV, a tube current of 75 mAs, and a slice thickness of 5 mm. Images were reconstructed utilizing a B60f filter, producing images with a slice thickness and an interslice gap of 1 mm for axial imaging. The CT images, formatted in DICOM, were loaded onto a computer workstation for segmentation into regions of interest (ROI), appear with suitable window levels and window widths. The delineation target comprised the adrenal glands of qualifying patients; due to the negative display of the images, we outlined the entire adrenal gland to extract features. The reports were verified for accuracy by a radiologist with a 5-year CT imaging interpretation experience. Two resident physicians, each with 2 years of experience, manually drafted two-dimensional (2D) ROIs, meticulously outlining each layer around the target adrenal gland profile to establish three-dimensional volumes of interest (VOIs) on non-enhanced CT images, subsequently reviewed by a chief physician. When delineating and reviewing the target areas, they were all unaware of whether the areas they were delineating or reviewing were lesions. For every scan, the extension SlicerRadiomics (from 3D Slicer 5.0.3, version aa418a5) was used, deriving from the Python-coded (version 3.9.10) PyRadiomics package (version 3.0.1), extracted an aggregate of 107 radiomic features (RFs).

### 2.3 RFs selection

**Figure 2:**
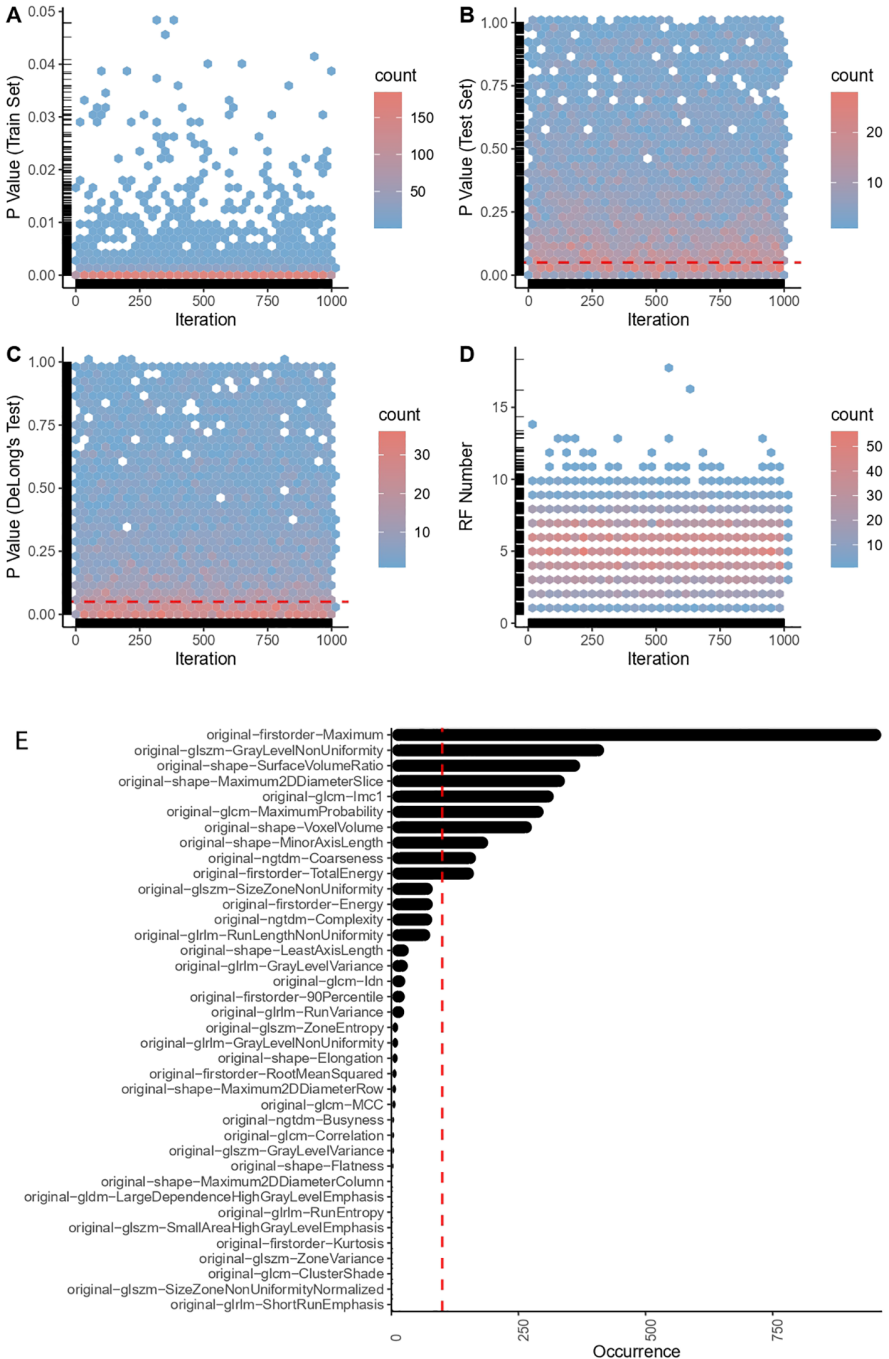
Radiomic feature engineering outcomes. **(A)** P-values in the train set. **(B)** P-values in the test set. **(C)** P-value of DeLong’s test. **(D)** Number of successfully captured RFs (radiomic features). **(E)** Top robust radiomic features in the final selection.

Predictive models were constructed based on RFs. To ensure their stability, an Intraclass Correlation Coefficient (ICC) examination was first deployed, discarding features with significant variability between two delineations to reduce errors resulting from manual outlining tasks. Subsequently, an iteratively designed algorithm specified in R programming language (version 4.1.3) conducted rigorous screenings on RFs in the derivation cohort (which had undergone ICC screening), as previously reported (30) (**Figure 2**). ICC test results are shown in **Supplementary Table 7**.

During each iteration, samples in the derivation cohort were randomly divided into training and test sets at ratios of 8:2, 7.5:2.5, 7:3, 6.5:3.5, 6:4, 5.5:4.5, 5:5 successively. On the training set, the ‘caret’ package (version 6.0–93) was initially employed to standardize data and filter out the zero variance RFs. Thereafter, the normality of each RF was tested for redundancy (Pearson correlation test was conducted if two RFs demonstrated a normal distribution, otherwise, a Spearman correlation test was performed). RFs that showed a correlation coefficient of over 0.9 and a P-value lower than 0.05 were deemed redundant and were excluded from subsequent processes (preserving only one non-redundant RF). Following that, the ‘coin’ package (version 1.4–2) was used to conduct a permutation test on RFs extracted from the redundancy testing. RFs showing no statistical difference (P < 0.05) between case and control patients were accordingly removed. Then, we leveraged the Least Absolute Shrinkage and Selection Operator (Lasso)—a multivariate algorithm that penalizes variables to minimize model error—via the ‘glmnet’ package (version 4.1–4) for further RFs selection and initial predictive modeling (incorporating Lasso penalty with the smallest mean squared error). Correspondingly, a mathematical formula was utilized to calculate a Radiomics Score (RS) for each sample:

*RS* = $\sum_{i=1}^{n}RF\;Value\;\times \;Penalized\;Coefficient\;$ (30)

Here, n refers to the number of RFs, RF_Value indicates the value of each RF, Penalized Coefficient pertains to each RF in the Lasso model with the smallest mean squared error (optimal weight assigned to each RF for prediction in the Lasso model), and Intercept embodies the constant term in the equation. On the test set, we used the means and standard deviations computed from the training set to standardize the test data, employing the aforementioned formula to calculate the RS for each sample. Eventually, employing the ‘pROC’ package (version 1.18.0), the performance and generalizability of the Lasso model were assessed via Receiver Operating Characteristic (ROC) curve analysis. If there was no statistical difference in ROC curves between the training and testing sets (DeLong test, P > 0.05), and a significant difference existed in RS between the two groups of case patients and control patients (permutation test, P < 0.05), RFs chosen for the RS formula were retained. This iteration was performed 1,000 times. RFs selected more than 100 times were employed for the final radiomic model construction and biomarker development. (**Figure 2**).

### 2.4 Radiomic model

Upon determining the most robust RFs, data within the derivation cohort was split again into training and test sets at a ratio of 6.5:3.5, enabling us to perform the final Machine Learning (ML) modeling. The training set data were standardization(normalized). Subsequently, six widely utilized ML algorithms, namely Ridge Regression, Lasso Regression, Logistic Regression (LR), Linear Discriminant Analysis (LDA), the K-Nearest Neighbors (KNN), and Support Vector Machines (SVM), were employed to train predictive models within the derivation cohort. Among these, Lasso and Ridge models adjusted their penalty terms through 10-fold cross-validation to pinpoint the penalty term which minimizes the mean squared error of the models. The LR model was trained using the default settings of the ‘stats’ package (version 4.1.3), and a nomogram was constructed with the ‘rms’ package (version 6.3–0). The LDA model was trained with the default settings of the ‘MASS’ package (version 7.3–55). The KNN model was trained with the ‘class’ package (version 7.3–20), its hyperparameter k adjusted from 1 to 50 to determine its optimum value. We employed a KNN training set for hyperparameter tuning, utilizing 10-fold cross-validation. Cross-validation was performed for each value of K, and the test set was reserved for the final performance assessment. For SVM, we divided the dataset into training, validation, and test sets. There was no data overlap between the validation and test sets to avoid data leakage. The training set was used for model training, the validation set for selecting hyperparameters and kernel functions, and the test set for the final performance evaluation. The ‘kernlab’ package (version 0.9–31) was adopted to train the SVM model and adjust the SVM kernel, covering a range of kernel functions such as “rbfdot,” “polydot,” “tanhdot,” “vanilladot,” “laplacedot,” “besseldot,” and “anovadot”. When assessing the performance of these AI models, we utilized methods identical to those employed in the iterative section.

### 2.5 Clinical-radiomic model

The highest performing model from the training was deployed to attribute a quantitative score to each adrenal CT image scan, termed the RS. Subsequently, we performed a univariate logistic regression (LR) on the RS and collected clinical information. Features with a P-value lower than 0.05 were included in a multivariate LR to construct an integrated radiomics-clinical model, which can calculate a composite score, i.e., the Clinical-Radiomics Score (Clin-Rad. Score), for each adrenal CT scan. In this study, gender, age, systolic pressure, diastolic pressure, hypertension duration, serum aldosterone, serum renin, renal function (serum uric acid, serum creatinine, eGFR), blood potassium, urine analysis (urine potassium, 24-hour urine aldosterone, urine protein/creatinine ratio), blood lipid levels (TG, TC, LDL, HDL), and echocardiography indices (ejection fraction, diastolic interventricular septum thickness) were included in the analysis. As for the small portion of missing data, they were filled by employing the mean, median, or mode imputation method depending on whether their data profile conforms to a normal or skewed distribution. For these clinical data, t-test and Mann-Whitney U test (U-test) were firstly employed to screen indicators with statistical differences between the two groups. Subsequently, univariate and multivariate analyses were used to further select meaningful clinical characteristics. Eventually, we would utilize the logistic regression method to construct a Clinical-Radiomics model with these meaningful clinical characteristics and RS combined.

We employed the Youden index to establish the thresholds of the RS and the Clin-Rad. Score, assisting in differentiating between lesional adrenal glands and normal adrenal glands. To assess the generalizability of the developed models, the ROC curves between the training and test sets were compared. The AUC, accuracy, and other performance indices, along with the DeLong test, were used to evaluate the diagnostic capacity of the selected features (biomarkers) within the derivation and temporal validation cohorts; the Hosmer-Lemeshow (HL) test served to appraise the goodness of fit of our models, and calibration curves were leveraged for gauging the precision of model predictions. Furthermore, the Decision Curve Analysis (DCA) was employed to estimate the clinical utility of the diagnostic tests or predictive models.

### 2.6 Statistical analysis

Categorical variables are presented in the form of counts (n) and percentages (%). For normally distributed data, continuous data are showcased as mean ± standard deviation, whereas data with skewed distribution are elucidated by the median (interquartile range). Dependent on the need, a variety of methodologies including normality test, variance analysis, Pearson’s correlation test, Spearman’s correlation test, two-tailed t-test, chi-squared test, Fisher’s exact test, Mann-Whitney U test, and Permutation test, were enlisted to statistically compare different groups when summarizing the clinical characteristics of research participants. Furthermore, a permutation test was implemented. In all tests presumed, a two-tailed p-value of lower than 0.05 was taken as indicative of statistical significance.

## 3 Results

### 3.1 Clinical features

The clinical features of the participants in the derivation and temporal validation cohorts are presented as depicted in **Supplementary Tables 8**, **9**. Within the derivation cohort, 120 patients diagnosed with PA — inclusive of 49 females (40.8%) and 71 males (59.2%). No significant variances in gender (P=0.10) and age (P=0.54) were observed between the experimental group and the control group. Likewise, key indicators such as height, weight, BMI, glomerular filtration rate, systolic pressure, diastolic pressure, duration of hypertension, echocardiographic ejection fraction, serum creatinine, urinary potassium, 24-hour urine potassium, HDL, LDL, and total cholesterol exhibited no statistical disparity between the control and experimental groups. Features discerned as disparate have been retained for univariate and multivariate analyses, as well as for the establishment of the terminal logistic regression model. In the temporal validation cohort, their age and gender also did not present any statistical difference.

### 3.2 RFs selection

Two physicians independently reviewed the transverse images of the adrenal glands (CT plain scan images), and eligible pictures were selected, as expounded in the method section. A sum of 107 RFs were extracted from each of these images using 3D Slicer. Following the appraisal through ICC tests—eliminating RFs that manifest significant variability due to manual delineation—an aggregate of 100 RFs was retained. This comprised 14 SHAPE RFs (14%), 18 FIRSTORDER RFs (18%), 24 GLCM RFs (24%), 12 GLDM RFs (12%), 14 GLRLM RFs (14%), 13 GLSZM RFs (13%), and 5 NGTDM RFs (5%) (**Supplementary Material 1**). A particularly devised robust RF selection iterative process was subsequently used to refine these RFs. During the iterations, a successful model was surmised to meet two criteria: 1). RFs in the test set demarcate significant disparateness between the experimental and control groups (using permutation tests, P < 0.05), and 2). A lack of significant discrepancies between the ROC curves of the training and test groups (via DeLong’s test, P > 0.50). Among a total of 1000 iterations, we constructed 7000 Lasso models; that is, seven models per iteration. RFs that appeared more than 100 times (10 RFs) were identified and selected for downstream analysis (**Figure 2**).

### 3.3 Development and validation of radiomic models

**Figure 3:**
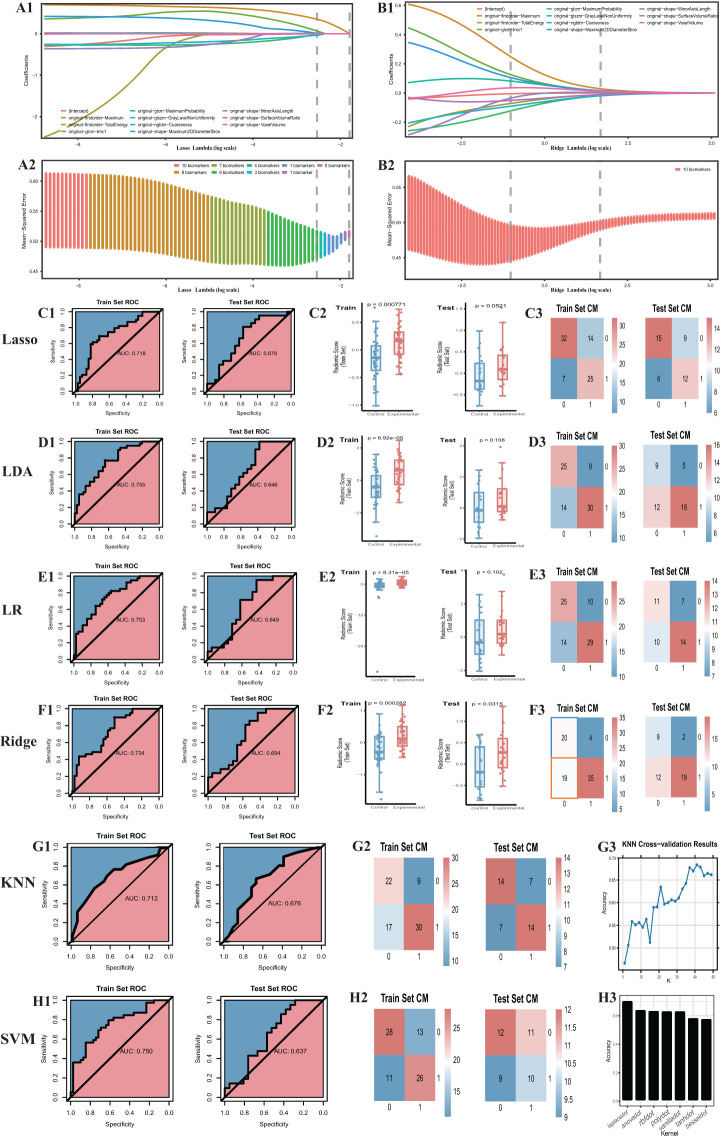
Outcome of machine learning models including Ridge, Lasso, LDA, LR, KNN, SVM. **(A)** Cross-validation of the lasso model. **(B)** Cross-validation of the ridge model. **(C1)** the ROC curve. The same to **(D1-H1)** LDA, LR, Ridge, KNN, SVM model. **(C2)** radiomic score comparison of the Lasso model (Box-plot of permutation test). The same to **(D2-F2)** LDA, LR, Ridge model. **(C3)** Confusion matrix. The same to **(D3-F3)** and **(G2-H2)** LDA, LR, Ridge, KNN, SVM model. **(G3)** Hyper-parameter tuning outcome of the KNN model. **(H3)** Hyperparameter tuning outcome of the SVM model. CM, confusion matrix; ROC, receiver operating characteristic.

Initially, we established four models: ridge regression, Lasso, LR, and LDA to quantitatively assign a RS to each adrenal cross-sectional image based on the most robust RFs. For ridge regression and Lasso models, we determined the optimal regularization penalty through tenfold cross-validation. The confusion matrices and ROC curves of the models, indicated in **Figures 3C1-3, F1-3**, presented accuracies of 70.5% and 73.1% and AUCs of 0.734 and 0.717, respectively. Subsequently, the RS for the cross-sectional adrenal scans was computed using ridge regression and Lasso models. Similarly, LR and LDA models were employed to score the adrenal transection image scans. Regrettably, in the training and test sets, permutation tests performed on the RS of the control and experimental groups within each set revealed that only the ridge regression model had a p-value less than 0.05 (training set: P=0.003; validation set: P=0.03); the permutation test p-values in the validation sets for the Lasso, LR, and LDA models were all greater than 0.05, indicating a failed modeling attempt. Additionally, we investigated two ML models that directly provided predictive probabilities for the experimental group data: KNN and SVM. For KNN, the hyperparameter k was set to 41, achieving the highest accuracy (66.7%) in the test set, with the corresponding AUC indicated in **Figure 3G1-3**. For SVM, we observed that an SVM model employing the Laplacian kernel achieved the highest accuracy (69.2%) in the test set (**Figure 3H1-3**). Therefore, the kernel function ultimately selected was laplacedot (**Figure 3**).

In summation, only three models were successful in modeling: ridge regression, KNN, and SVM. Detailed performance evaluations are presented in **Table 1**. Additionally, regarding ridge regression, we compared the RS ROC curves of the derivation cohort and the temporal validation cohort, the area under the curve (AUC) of which were 0.719 and 0.677, respectively (refer to **Supplementary Material 4**). The Delong test (P=0.64) suggests there is no significant difference in their predictive capacity. However, their permutation tests (derivation cohort P<0.05, temporal validation cohort P=0.02) indicate good differentiation between the experimental and control groups within the derivation cohort and the temporal validation cohort. This implies that the RS value also holds the potential to serve as a diagnostic and predictive biomarker.

**Table 1:** Performance of eight radiomics models.

Name	KNN	Lasso	LDA	LR	Ridge	SVM
Partition Rate in Train Set	65%	65%	65%	65%	65%	65%
Comparison Method of ROC	DeLong’s test	DeLong’s test	DeLong’s test	DeLong’s test	DeLong’s test	DeLong’s test
P Value	0.857	0.687	0.294	0.321	0.690	0.286
Train Set:						
Train AUC	0.712	0.718	0.755	0.753	0.734	0.750
Sensitivity (TPR)	0.564	0.821	0.641	0.641	0.513	0.718
Specificity (TNR)	0.769	0.641	0.769	0.744	0.897	0.667
Accuracy (ACC)	0.667	0.731	0.705	0.692	0.705	0.692
Error Rate (ER)	0.333	0.269	0.295	0.308	0.295	0.308
Recall	0.564	0.821	0.641	0.641	0.513	0.718
F1	0.629	0.704	0.723	0.707	0.753	0.700
Kappa	0.333	0.462	0.410	0.385	0.410	0.385
Test Set:						
Test AUC	0.676	0.676	0.646	0.649	0.694	0.637
Sensitivity (TPR)	0.667	0.714	0.429	0.524	0.429	0.571
Specificity (TNR)	0.667	0.571	0.762	0.667	0.905	0.476
Accuracy (ACC)	0.667	0.643	0.595	0.595	0.667	0.524
Error Rate (ER)	0.333	0.357	0.405	0.405	0.333	0.476
Recall	0.619	0.714	0.429	0.524	0.429	0.571
F1	0.667	0.615	0.653	0.622	0.731	0.545
Kappa	0.333	0.286	0.190	0.190	0.333	0.048

*AUC, area under the curve; LR, logistic regression; LDA, linear discriminant analysis; KNN, k-nearest neighbors; SVM, support vector machine; TPR, True Positive Rate; TNR, True Negative Rate.

### 3.4 Clinical-radiomic model construction

Among the models that were successful, the ridge regression model demonstrated notable discriminative performance in both training and test sets. Its difference between the AUC of the training set and the test set was minimal, with the training set’s AUC ranking second, and the test set’s AUC securing the top position. Consequently, the RS, derived from the ridge regression model, was selected as the optimal radiomics base biomarker for subtype diagnosis, to be employed in the construction of the clinical-imaging radiomics model. Based on the Youden index, we determined the cutoff for the RS to be -0.28, with a sensitivity of 0.513 and specificity of 0.897 (**Table 1**). RS higher than this threshold signifies that the corresponding adrenal gland may potentially be a lesional one.

To enhance the performance of the RS, we integrated it with clinical features using the conventional logistic regression (LR) algorithm. Based on the type and normality of clinical data, we carried out Chi-square tests, t-tests, and rank-sum tests, initially identifying seven clinical features - K^+^(Min.), Echo-IVSd, RF-UA, 24h AE(Max.), Renin, Aldo-max, BL-TG - which presented differences between the control and experimental groups. Prior to model construction, we utilized univariate and multivariate regression analyses to screen the features. During the first round of univariate and multivariate analyses, we noted that the P value for Renin in univariate analysis was greater than 0.05. Simultaneously, P values for Aldo-max, BL-TG in multivariate analysis exceeded 0.05 (**Supplementary Material 5**). Owing to the smaller sample size for this study and the necessity to streamline features, we decided to exclude these. Herein, the RS was validated as meaningful in the new model. Consequently, four clinical features were selected for the construction of the clinical-imaging radiomics model: K^+^(Min.), Echo-IVSd, RF-UA, 24h AE(Max.). After conducting a second round of univariate and multivariate regression with these four clinical features and RS, all P values were found to be less than 0.05, indicating their high relevance with the outcome. The results of univariate and multivariate LR analyses for RS and clinical features are presented in **Supplementary Material 5**. Compared to the radiomics model (ridge regression), the integration of clinical data enhanced the performance of the AI model; the AUC values for the derivation cohort were 0.868 and those for the temporal validation cohort were 0.853. The performance of this integrated Clinical-Radiomics model surpassed that of the single radiomics model (**Table 2**). Furthermore, the Delong test P value for the model was 0.18, greater than 0.05, and HL test P values were all above 0.05. Calibration curves were plotted to evaluate fitting capabilities for both cohorts, and DCA was used to assess clinical utility. We calculated a comprehensive score for each adrenal cross-sectional image, termed as the ‘Clin-Rad. Score’, which we propose as a novel biomarker for PA patients and verified in a temporal validation cohort.

**Table 2:** Performance of clinical-radiomic model.

Data	AUC (95%CI)	Accuracy (95%CI)	Sensitivity (95%CI)	Specificity (95%CI)	PPV (95%CI)	NPV (95%CI)	Cut off
Derivation	0.868 (0.804-0.932)	0.783 (0.699-0.853)	0.700 (0.584 - 0.816)	0.867 (0.781 - 0.953)	0.840 (0.738 - 0.942)	0.743 (0.640 - 0.845)	0.440
Validation	0.853 (0.750-0.956)	0.760 (0.618-0.869)	0.720 (0.544 - 0.896)	0.800 (0.643 - 0.957)	0.783 (0.614 - 0.951)	0.741 (0.575 - 0.906)	0.440

*AUC, area under the curve; PPV, Positive Predictive Value; NPV, Negative Predictive Value.

**Figure 4:**
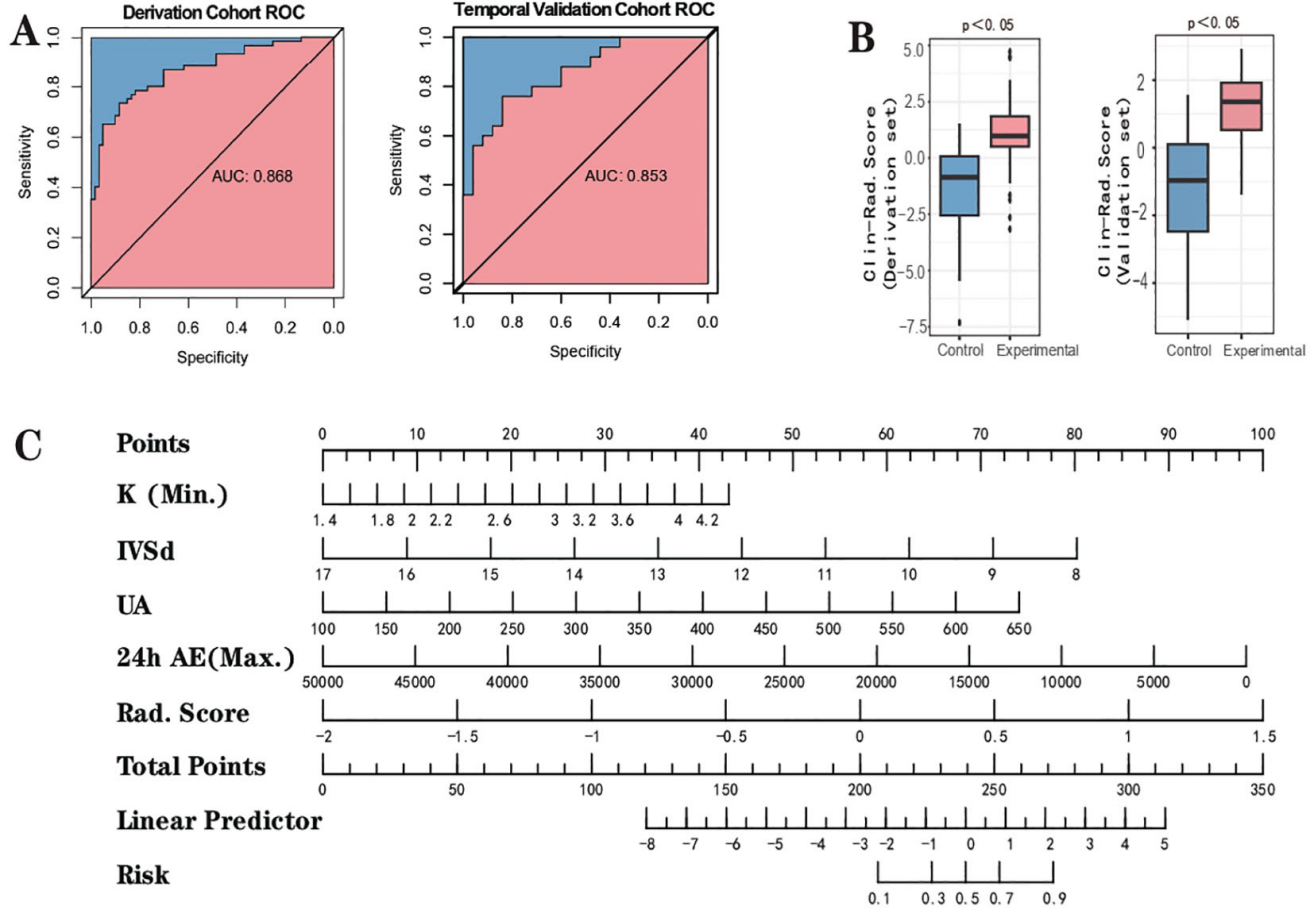
Outcome of clinical-radiomic model. **(A)** the ROC curve. **(B)** Clinical-Radiomic score comparison of the Clinical-Radiomics model (Box-plot of permutation test). **(C)** Nomogram.

The cut-off value for the Clin-Rad. Score is determined to be 0.63, exhibiting a sensitivity of 88.3% and a specificity of 73.3% in the derivation cohort (**Supplementary Material 5**). Meanwhile, in the temporal validation cohort, it illustrated a sensitivity of 84.0% coupled with a specificity of 72.0%. To achieve a higher specificity and thereby minimize the risk of misdiagnosis, we adjusted the cut-off value to 0.440. At this value, the best specificity is obtained while maintaining a satisfactory sensitivity. The sensitivity and specificity of the derivation cohort have been transformed to 70.0% and 86.7%, respectively, while the sensitivity and specificity of the validation cohort have been transformed to 72.0% and 80.0%, respectively (**Table 2**). The Clin-Rad. Score demonstrated independent predictive power. According to the permutation test results (derived cohort P<0.05, temporal validation cohort P<0.05), it indeed distinguishes between the experimental group and the control group (P<0.05). The Delong test for both cohorts (P=0.81) implies that there is no statistically significant discrepancy in the predictive efficacy between the two ROC curves. we constructed a nomogram for Clin-Rad. Score, readily facilitating its use in clinical practice. The HL test (derived cohort P=0.083, temporal validation cohort P=0.56) and the calibration curve suggest the model’s fitting capability is acceptable (**Supplementary Material 4**). The DCA curve reveals the changes in the net benefit of the model at different risk thresholds, indicating that the established model shows good clinical utility. When the threshold exceeds 0.20, the model begins to exhibit its superiority (**Supplementary Material 4**; **Figure 4**).

## 4 Discussion

In this investigation, we retrospectively collected cross-sectional adrenal scans from 144 patients diagnosed with PA. RFs were extricated from these scans and rigorously sifted through a dedicated iterative process to recognize the most robust and stable features (30). A selection of 10 RFs along with 6 ML techniques were employed to construct the radiomics model, and subsequently calculate the RS. Concurrently, the clinical indicators of these 170 patients underwent rigorous statistical evaluation, culminating in the amalgamation of four unique clinical features and the Ridge Regression model’s RS to construct the composite clinical-radiomics model. From this, we successfully developed and validated two quantitative diagnostic markers based on radiomics, namely the RS and the Clin-Rad. Score. These will be utilized for automatic prediction of cross-sectional negative adrenals in the subtype diagnosis of PA, essentially serving as forecasting variables of the Artificial Intelligence (AI) model. Although erstwhile exploration pertaining to radiomics modeling for subtype diagnosis of PA is documented (28), to our knowledge, we are the first to extract the peculiar subtype of PA characterized by cross-sectional negative adrenal lesions among the myriads of PA subtypes. We utilized radiomics methods to construct an AI model for the development of PA diagnostic markers and automated prediction of this unique subtype.

The subtype diagnosis of PA is presently a central preoccupation spanning domains of endocrinology, cardiovascular medicine, urology, and imaging science at home and abroad. Within the architecture and evolution of clinical predictive models or standards, numerous scholars across the globe have made enthusiastic forays. These models or standards are primarily based on general data, outcomes of laboratory examinations, and results from imaging studies. As early as 2008, the team of Mulatero tested the sensitivity and specificity of CT scanning, as well as the usefulness of clinical standards (renin, aldosterone) in differentiating between aldosterone-producing adenoma (APA) and bilateral adrenal hyperplasia (BAH). However, the discrimination does not meet expectations, which emphasized the significance of AVS (31). Küpers et al. proposed a clinical prediction score (CPS) for unilateral PA, rooted in clinical and imaging indicators such as typical Conn’s adenoma, hypokalemia, and eGFR (32). Sze and colleagues made slight modifications to CPS and Radiomics Grading Score (RGS). Their findings suggest that these revised scores, or their combination with RGS, do not improve predictive performance (33). Zhang Y and others observed that upon adjustment of the model proposed by Küpers et al., features including urinary aldosterone levels, history of hypokalemia, and the presence of typical unilateral adenomas larger than 1 cm in diameter result in a 90.5% diagnostic specificity (22). Kocjan et al. found a combination of serum potassium ≥3.5 mmol/L, post-SIT aldosterone <18 ng/dL, and no bilateral tumors or their presence on CT imaging acted as predictors of bilateral PA with 100% specificity (34). This methodology is the maiden one to demonstrate that clinical predictive standards can accurately identify patients with non-lateralized AVS (bilateral dominant secretion). Researchers at Ruijin Hospital, Shanghai, modeled a nomogram to predict the probability of IHA, comprising BMI, blood potassium level, and adrenal CT findings, yielding sensitivity and specificity exceeding 85% (3). Jacopo Burrello and colleagues developed a predictive model for the diagnosis of PA subtypes utilizing clinical and biochemical characteristics of patients and geared towards the prognosis of both unilateral (LPA) and bilateral (BPA) diseases (35). The model variables included: Aldosterone at screening, Aldosterone post-confirmatory test, Lowest potassium value, Presence/absence of nodules at CT scanning, Nodule diameter at CT scanning, and CT scanning findings. Mansour and colleagues endeavored to enhance the subtype prediction of PA by directly amalgamating clinical parameters and RFs based on CT imaging. This included predictions for unilateral left, unilateral right, as well as bilateral dominant secretion. Regrettably, the potentials diagnosed from their works to distinguish lesion locations were constrained, yielding a mere 0.56 area under the curve (AUC) for the consolidated radiomics model (28). The team of Po-Ting Chen achieved subtype prediction in PA patients through a two-stage model construction, the first stage being the development of a model to differentiate between unilateral or bilateral PA, and the second being the design of a model to determine the dominant side in patients with unilateral PA (29).

We perceive that the aforementioned models or scores harbor the following issues: 1) Clinical models demonstrate a decent predictive accuracy for bilateral PA (BPA) or conspicuous unilateral aldosterone-producing adenoma (APA). However, they struggle with the detailed localization of unilateral non-adenoma lesion (left or right), making it difficult to circumvent AVS when a unilateral subtype prediction is made. 2) Regarding papers that enriched RFs (28, 29), their strength lies in augmenting the volume of data and characteristics intrinsic to the unilateral adrenal gland, enabling further precision in predictive localization, i.e., direct prediction of left or right lesions. Nevertheless, they might overlook the diversity present in the morphology of a single adrenal gland from CT images. Such morphological diversity could interfere with the comparability of extracted RFs, which will be discussed in detail in the context of strengths and limitations of subsequent research. 3) The sample sizes across these studies were relatively small, likely due to AVS being somewhat uncommon and the patient count being limited in each center. Moreover, the generalizability of each model requires further substantiation.

Our study identified significant statistical differences in serum potassium (lowest), aldosterone (highest), renin, 24-hour urinary aldosterone excretion (highest), interventricular septal thickness at end-diastole via echocardiogram, uric acid, and lipids (TG) between the experimental and control groups. This suggests heterogeneity in the levels of aldosterone secretion among the subtypes of PA under investigation. From the perspective of monism, differences in multiple clinical features between the control and experimental groups could be attributed to serum aldosterone levels. As is widely acknowledged, the level of aldosterone naturally influences serum potassium and urinary aldosterone, with elevated serum aldosterone suppressing renin levels while promoting potassium excretion, and concurrently escalating urinary aldosterone excretion (9). Research conducted by Silvia Monticone et al. posited that, compared to patients with essential hypertension (EH), those with PA stand a higher risk for left ventricular hypertrophy, seemingly associated with excessive aldosterone secretion (36). Animal model research underscores that excessive aldosterone can instigate vascular inflammation, interstitial fibrosis in various organs and tissues, and endothelial dysfunction, which could potentially account for aldosterone-induced ventricular remodeling (37, 38). Whether different levels of aldosterone result in varying degrees of left ventricular hypertrophy is a question worthwhile of future exploration. A study by Worapaka Manosroi et al. observed that compared to patients with EH, TG levels were lower in patients with PA (39). The relationship between serum aldosterone levels and serum uric acid levels appears infrequently in the literature, but one dated publication describes that the lower SUA (compared to EH) in PA is due to the inhibition of reabsorption in the proximal renal tubules and/or an increase in uric acid secretion, which is associated with the so-called “escape phenomenon” (40). In our study, the level of aldosterone in the control group was higher compared to the test group, whereas the TG and uric acid levels were lower in the control group, which aligns with previous research. The underlying mechanisms warrant further exploration.

Due to the rarity of patients undergoing adrenalectomy, with the majority opting for medical treatment, it is challenging to obtain their pathological reports. The common pathological classifications for PA include APA, aldosterone-producing nodules, aldosterone-producing micronodules (APMs) and adrenocortical hyperplasia (41). Aldosterone-producing cell clusters (APCCs) may represent a transitional state between pathological adrenal cortical tissue and normal tissue, and are considered a special type of cell cluster. Japanese researcher Iwahashi has hypothesized that APCCs are pathogenic factors for IHA and precursors to APAs through single-cell analysis (42). We speculate that the pathological changes in lesion adrenal glands with normal imaging findings may be minor adrenal lesions or hyperplasia. Japanese researchers, including Yuto Yamazaki, have analyzed a group of Cross-Sectional Image-Negative adrenal glands in hyperaldosteronism. They found that these special types of adrenal glands were classified as multiple adrenocortical micronodules (MN; n = 13) or diffuse hyperplasia of the zona glomerulosa (ZG; n = 12) based on histopathological evaluation and CYP11B2 immunohistochemistry (14). We hypothesize that these subtle pathological changes (micro nodules or hyperplasia) may be recorded by imaging examinations but are not recognizable to the naked eye, leading to their classification as morphologically normal adrenal glands. Therefore, this study employs radiomics and machine learning models to enhance the identification and extraction of minute and unique features through computer and AI techniques, aiming to identify lesion adrenal glands. This forms the basis of our research hypothesis. CT has a diagnostic capability of 0 for this type of adrenal gland, whereas our study, utilizing machine learning and radiomics, has achieved a specificity greater than 0.8 and a sensitivity greater than 0.7 for diagnosing this special type of adrenal gland and subtype of PA, demonstrating potential for clinical application.

One of the strengths of our research is the meticulous quantification at each step, ranging from the initial selection of the ROI to the final determination of the AI model coefficients. This permits our radiomics-based AI model to be easily validated and further trained in different centers or nations in future undertakings. Furthermore, we utilized a distinctive feature engineering and iterative process, ensuring the robustness of the derived RFs and maintaining consistency and fairness of the final ML model input features. This method has previously been employed and confirmed for its reliable performance in earlier studies (30). Successful efficacious models can save patients’ finances and time, in addition to attenuating the discomfort caused by invasive procedures. The integration of radiomics techniques and AI models into CT scanners, allowing for immediate prognostic outcome output, would facilitate physicians in making judicious decisions, particularly in lower-tier hospitals lacking AVS technology. Another merit of our study lies in our controlling the study population and scope; the adrenals under investigation were all cross-sectional negative adrenals, optimizing model performance to the maximum extent. The subtype diagnosis of PA is intricate. While AVS simplifies it into unilateral (left, right) and bilateral dominant secretions, the internist, prior to conducting the AVS, needs to consider whether a cross-sectional negative adrenal could potentially be the dominant secretion, whether a cross-sectional positive adrenal could potentially be a non-functioning adenoma, and differentiate the various cross-sectional positive adrenal morphologies: thickened, nodular, adenoma, which are the reasons for the inconsistency between AVS and radiological examinations (43). Therefore, PA patients’ lesional adrenals cannot be plainly identified based on clinical blood indicators and imaging data. If we overlook the disparity in adrenal imaging morphologies and solely categorize all adrenal images (including cross-sectional negative and positive) as lesion side (experimental group) and non-lesion side (control group) based on the AVS results for model prediction, it will result in the presence of both normal and abnormal adrenal glands in both the experimental and control groups. The potential benefit is the possibility of obtaining a larger sample size, but there is no doubt that this will reduce the comparability of imaging images and impair the diagnostic efficiency of prediction models (28, 29).

There are some limitations within our study that merit consideration. They primarily include the following aspects: 1) Constrained by the sample size, this resulted in room for improvement in model precision and a lack of generalization capability. Future research could optimize the model by integrating a larger dataset. 2) In addition to the limited sample size, this study was also conducted in a single center, which may restrict the external validity of the findings. Single-center studies may be subject to specific population characteristics and local practice patterns, limiting broader applicability. Future research should consider multicenter collaboration to enhance generalizability. 3) The refinement of subgroups could potentially enhance precision and generalization. For instance, there is a specific patient type within the experimental group: patients with bilateral adrenal CT images showing no abnormalities, but their results of AVS indicate unilateral dominant secretion. Such patients only constitute around 11 individuals within the experimental group. If a larger sample size were available, we could extract their features from bilateral adrenal images individually, and then perform modeling (subgroup analysis). The resultant radiomic models would not require the addition of clinical features and could be directly applied to clinical predictions for this specific group of patients as the control and experimental groups, derived from the same cohort, bear similar clinical features. The significance of subgroup division lies in the fact that each individual possesses two adrenal glands, and our study only includes those with a single side of negative adrenal images. If we could control the consistency of non-research adrenals within the test and control groups as much as possible, we believe that our model’s performance may see further improvement, and its generalizability might be enhanced concurrently. A prerequisite, of course, is the availability of more samples. 4) The area under the curve (AUC) of our radiomics model is not particularly high. We believe one reason is the inherent characteristics of the adrenal glands with negative imaging findings in our study. These glands often have subtle lesions that are extremely difficult to differentiate, and CT has virtually no diagnostic capability for such adrenal glands. This intrinsic limitation could naturally lead to model failure. Additionally, due to the minuscule size of the lesions, which are barely detectable on imaging, we had to delineate the entire adrenal gland during region of interest (ROI) delineation. This inevitably included a large amount of normal tissue, further complicating meaningful feature extraction and model construction. Nevertheless, by relying solely on imaging data without incorporating clinical indicators, we achieved an AUC of approximately 0.7. To some extent, this still holds preliminary screening significance and provides inspiration for future research. The permutation test revealed a significant difference in radiomics scores between the experimental and control groups in ridge model (p < 0.05). While we certainly aspire to develop a model capable of perfectly distinguishing lesions, there is still a considerable gap to bridge given the current state of scientific and technological advancements. Moving forward, we can explore multi-modal data modeling and incorporate more comprehensive information to gradually approach this ideal goal. 5) An active exploration of novel modeling methods is essential. With the ubiquitous application and rapid evolution of AI, increasingly novel methodologies are being promoted, which form the focus of ensuing work and learning for our research group. Future work necessitates further validation through prospective studies.

In summary, the diagnostic markers for PA and the clinical-radiomic models developed in our study hold considerable potential for future translational research. For the first time, we provide an objective quantifiable metric based on AI and ML models for the recognition of a specific subtype of PA, namely, cross-sectional negative lesional adrenals. This offers an innovative approach and solution for the eventual realization of automated subtype diagnosis of all types of PA.

## Data Availability

The original contributions presented in the study are included in the article/**Supplementary Material**. Further inquiries can be directed to the corresponding author.

## References

[1] Mullen N Curneen J Donlon PT Prakash P Bancos I Gurnell M. Treating primary aldosteronism-induced hypertension: novel approaches and future outlooks. Endocr Rev. (2023) 45:125–70. doi: 10.1210/endrev/bnad026, PMID: 37556722 PMC10765166

[2] Turcu AF Yang J Vaidya A. Primary aldosteronism - a multidimensional syndrome. Nat Rev Endocrinol. (2022) 18:665–82. doi: 10.1038/s41574-022-00730-2, PMID: 36045149

[3] Xiao L Jiang Y Zhang C Jiang L Zhou W Su T. A novel clinical nomogram to predict bilateral hyperaldosteronism in Chinese patients with primary aldosteronism. Clin Endocrinol (Oxf). (2019) 90:781–8. doi: 10.1111/cen.13962, PMID: 30820995

[4] Monticone S Burrello J Tizzani D Bertello C Viola A Buffolo F. Prevalence and clinical manifestations of primary aldosteronism encountered in primary care practice. J Am Coll Cardiol. (2017) 69:1811–20. doi: 10.1016/j.jacc.2017.01.052, PMID: 28385310

[5] Brown JM Siddiqui M Calhoun DA Carey RM Hopkins PN Williams GH. The unrecognized prevalence of primary aldosteronism: A cross-sectional study. Ann Intern Med. (2020) 173:10–20. doi: 10.7326/M20-0065, PMID: 32449886 PMC7459427

[6] Zennaro M-C Boulkroun S Fernandes-Rosa FL. Pathogenesis and treatment of primary aldosteronism. Nat Rev Endocrinol. (2020) 16:578–89. doi: 10.1038/s41574-020-0382-4, PMID: 32724183

[7] Endocrinology CS of. Expert consensus on the diagnosis and treatment of primary aldosteronism (2020). Chin J Endocrinol Metab. (2020) 36:727–36. doi: 10.3760/cma.j.cn311282-20200615-00444

[8] Reincke M Bancos I Mulatero P Scholl UI Stowasser M Williams TA. Diagnosis and treatment of primary aldosteronism. Lancet Diabetes Endocrinol. (2021) 9:876–92. doi: 10.1016/S2213-8587(21)00210-2, PMID: 34798068

[9] Funder JW Carey RM Mantero F Murad MH Reincke M Shibata H. The management of primary aldosteronism: case detection, diagnosis, and treatment: an endocrine society clinical practice guideline. J Clin Endocrinol Metab. (2016) 101:1889–916. doi: 10.1210/jc.2015-4061, PMID: 26934393

[10] Song Y Yang S He W Hu J Cheng Q Wang Y. Confirmatory tests for the diagnosis of primary aldosteronism: A prospective diagnostic accuracy study. Hypertension. (2018) 71:118–24. doi: 10.1161/HYPERTENSIONAHA.117.10197, PMID: 29158354

[11] Naruse M Murakami M Katabami T Kocjan T Parasiliti-Caprino M Quinkler M. International multicenter survey on screening and confirmatory testing in primary aldosteronism. Eur J Endocrinol. (2023) 188:lvac002. doi: 10.1093/ejendo/lvac002, PMID: 36726325

[12] Zuo R Liu S Xu L Pang H. Key to the treatment of primary aldosteronism in secondary hypertension: subtype diagnosis. Curr Hypertens Rep. (2023) 25:471–80. doi: 10.1007/s11906-023-01269-x, PMID: 37787864

[13] Scholl UI. Genetics of primary aldosteronism. Hypertension. (2022) 79:887–97. doi: 10.1161/HYPERTENSIONAHA.121.16498, PMID: 35139664 PMC8997684

[14] Yamazaki Y Nakamura Y Omata K Ise K Tezuka Y Ono Y. Histopathological classification of cross-sectional image-negative hyperaldosteronism. J Clin Endocrinol Metab. (2017) 102:1182–92. doi: 10.1210/jc.2016-2986, PMID: 28388725 PMC5460723

[15] Omura M Sasano H Saito J Yamaguchi K Kakuta Y Nishikawa T. Clinical characteristics of aldosterone-producing microadenoma, macroadenoma, and idiopathic hyperaldosteronism in 93 patients with primary aldosteronism. Hypertens Res. (2006) 29:883–9. doi: 10.1291/hypres.29.883, PMID: 17345788

[16] Hu J Xu T Shen H Song Y Yang J Zhang A. Accuracy of gallium-68 pentixafor positron emission tomography–computed tomography for subtyping diagnosis of primary aldosteronism. JAMA Netw Open. (2023) 6:e2255609. doi: 10.1001/jamanetworkopen.2022.55609, PMID: 36795418 PMC9936343

[17] Zheng Y Long T Peng N Zhen M Ye Q Zhang Z. The value of targeting CXCR4 with 68Ga-pentixafor PET/CT for subtyping primary aldosteronism. J Clin Endocrinol Metab. (2023) 109:171–82. doi: 10.1210/clinem/dgad421, PMID: 37477496

[18] Lenders JWM Eisenhofer G Reincke M. Subtyping of patients with primary aldosteronism: an update. Horm Metab Res. (2017) 49:922–8. doi: 10.1055/s-0043-122602, PMID: 29202492

[19] Kempers MJE. Systematic review: diagnostic procedures to differentiate unilateral from bilateral adrenal abnormality in primary aldosteronism. Ann Intern Med. (2009) 151:329. doi: 10.7326/0003-4819-151-5-200909010-00007, PMID: 19721021

[20] Zhou Y Wang D Jiang L Ran F Chen S Zhou P. Diagnostic accuracy of adrenal imaging for subtype diagnosis in primary aldosteronism: systematic review and meta-analysis. BMJ Open. (2020) 10:e038489. doi: 10.1136/bmjopen-2020-038489, PMID: 33384386 PMC7780716

[21] Rossi GP Barisa M Allolio B Auchus RJ Amar L Cohen D. The Adrenal Vein Sampling International Study (AVIS) for identifying the major subtypes of primary aldosteronism. J Clin Endocrinol Metab. (2012) 97:1606–14. doi: 10.1210/jc.2011-2830, PMID: 22399502

[22] Zhang Y Niu W Zheng F Zhang H Zhou W Shen Z. Identifying unilateral disease in Chinese patients with primary aldosteronism by using a modified prediction score. J Hypertens. (2017) 35:2486–92. doi: 10.1097/HJH.0000000000001488, PMID: 28708774 PMC5673302

[23] Mayerhoefer ME Materka A Langs G Häggström I Szczypiński P Gibbs P. Introduction to radiomics. J Nucl Med. (2020) 61:488–95. doi: 10.2967/jnumed.118.222893, PMID: 32060219 PMC9374044

[24] Yuan H Kang B Sun K Qin S Ji C Wang X. CT-based radiomics nomogram for differentiation of adrenal hyperplasia from lipid-poor adenoma: an exploratory study. BMC Med Imaging. (2023) 23:4. doi: 10.1186/s12880-022-00951-x, PMID: 36611159 PMC9826591

[25] Zheng Y Liu X Zhong Y Lv F Yang H. A preliminary study for distinguish hormone-secreting functional adrenocortical adenoma subtypes using multiparametric CT radiomics-based machine learning model and nomogram. Front Oncol. (2020) 10:570502. doi: 10.3389/fonc.2020.570502, PMID: 33117700 PMC7552922

[26] Chen P-T Chang D Liu K-L Liao W-C Wang W Chang C-C. Radiomics utilization to differentiate nonfunctional adenoma in essential hypertension and functional adenoma in primary aldosteronism. Sci Rep. (2022) 12:8892. doi: 10.1038/s41598-022-12835-9, PMID: 35614110 PMC9132956

[27] Zhang B Zhang H Li X Jin S Yang J Pan W. Can radiomics provide additional diagnostic value for identifying adrenal lipid-poor adenomas from non-adenomas on unenhanced CT? Front Oncol. (2022) 12:888778. doi: 10.3389/fonc.2022.888778, PMID: 35574405 PMC9102986

[28] Mansour N Mittermeier A Walter R Schachtner B Rudolph J Erber B. Integration of clinical parameters and CT-based radiomics improves machine learning assisted subtyping of primary hyperaldosteronism. Front Endocrinol. (2023) 14:1244342. doi: 10.3389/fendo.2023.1244342, PMID: 37693351 PMC10484561

[29] Chen P-T Li P-Y Liu K-L Wu V-C Lin Y-H Chueh JS. Machine learning model with computed tomography radiomics and clinicobiochemical characteristics predict the subtypes of patients with primary aldosteronism. Acad Radiol. (2024) 31:1818–27. doi: 10.1016/j.acra.2023.10.015, PMID: 38042624

[30] Lu A Li K Guo S Zhang X Su G Yang P. Development and validation of novel retina biomarkers and artificial intelligence models for Behçet disease uveitis prediction. Biomed Signal Process Control. (2024) 94:106271. doi: 10.1016/j.bspc.2024.106271

[31] Mulatero P Bertello C Rossato D Mengozzi G Milan A Garrone C. Roles of clinical criteria, computed tomography scan, and adrenal vein sampling in differential diagnosis of primary aldosteronism subtypes. J Clin Endocrinol Metab. (2008) 93:1366–71. doi: 10.1210/jc.2007-2055, PMID: 18198224

[32] Küpers EM Amar L Raynaud A Plouin P-F Steichen O. A clinical prediction score to diagnose unilateral primary aldosteronism. J Clin Endocrinol Metab. (2012) 97:3530–7. doi: 10.1210/jc.2012-1917, PMID: 22918872

[33] Sze WCC Soh LM Lau JH Reznek R Sahdev A Matson M. Diagnosing unilateral primary aldosteronism - comparison of a clinical prediction score, computed tomography and adrenal venous sampling. Clin Endocrinol (Oxf). (2014) 81:25–30. doi: 10.1111/cen.12374, PMID: 24274335

[34] Kocjan T Janez A Stankovic M Vidmar G Jensterle M. A new clinical prediction criterion accurately determines A subset of patients with bilateral primary aldosteronism before adrenal venous sampling. Endocr Pract. (2016) 22:587–94. doi: 10.4158/EP15982.OR, PMID: 26789347

[35] Burrello J Burrello A Pieroni J Sconfienza E Forestiero V Rabbia P. Development and validation of prediction models for subtype diagnosis of patients with primary aldosteronism. J Clin Endocrinol Metab. (2020) 105:dgaa379. doi: 10.1210/clinem/dgaa379, PMID: 32561919

[36] Monticone S D’Ascenzo F Moretti C Williams TA Veglio F Gaita F. Cardiovascular events and target organ damage in primary aldosteronism compared with essential hypertension: a systematic review and meta-analysis. Lancet Diabetes Endocrinol. (2018) 6:41–50. doi: 10.1016/S2213-8587(17)30319-4, PMID: 29129575

[37] Blasi ER Rocha R Rudolph AE Blomme EAG Polly ML McMahon EG. Aldosterone/salt induces renal inflammation and fibrosis in hypertensive rats. Kidney Int. (2003) 63:1791–800. doi: 10.1046/j.1523-1755.2003.00929.x, PMID: 12675855

[38] Nishizaka MK Zaman MA Green SA Renfroe KY Calhoun DA. Impaired endothelium-dependent flow-mediated vasodilation in hypertensive subjects with hyperaldosteronism. Circulation. (2004) 109:2857–61. doi: 10.1161/01.CIR.0000129307.26791.8E, PMID: 15173035

[39] Manosroi W Phudphong P Atthakomol P Phimphilai M. The differences of serum lipid profiles between primary aldosteronism and essential hypertension: a meta-analysis and systematic review. BMC Endocr Disord. (2022) 22:217. doi: 10.1186/s12902-022-01135-y, PMID: 36045354 PMC9429522

[40] Namba M Kikuchi K Komura H Suzuki S Satoh N Ohtomo T. Study on uric acid metabolism in patients with primary aldosteronism. Nihon Naibunpi Gakkai Zasshi. (1992) 68:51–61. doi: 10.1507/endocrine1927.68.1_51, PMID: 1541367

[41] Williams TA Reincke M. Pathophysiology and histopathology of primary aldosteronism. Trends Endocrinol Metab. (2022) 33:36–49. doi: 10.1016/j.tem.2021.10.002, PMID: 34743804

[42] Iwahashi N Umakoshi H Seki T Gomez-Sanchez CE Mukai K Suematsu M. Characterization of aldosterone-producing cell cluster (APCC) at single-cell resolution. J Clin Endocrinol Metab. (2022) 107:2439–48. doi: 10.1210/clinem/dgac394, PMID: 35796577 PMC9387688

[43] Aono D Kometani M Karashima S Usukura M Gondo Y Hashimoto A. Primary aldosteronism subtype discordance between computed tomography and adrenal venous sampling. Hypertens Res. (2019) 42:1942–50. doi: 10.1038/s41440-019-0310-y, PMID: 31409918

