# Supplementary material for: Development and validation of prediction models for special subtype of primary aldosteronism: patients with negative adrenal CT imaging
Source: Front Endocrinol (Lausanne). 2025 Jul 11;16:1563748. doi: 10.3389/fendo.2025.1563748 (PMC12289499; doi:10.3389/fendo.2025.1563748)
Supplement: Supplementary file 1 [file DataSheet1.pdf]

Supplementary material4: Calibration Curve, HL test, DCA, RS AUC, Permutation test, DELONG test

①Calibration Curve of Clinical-Radiomics modle

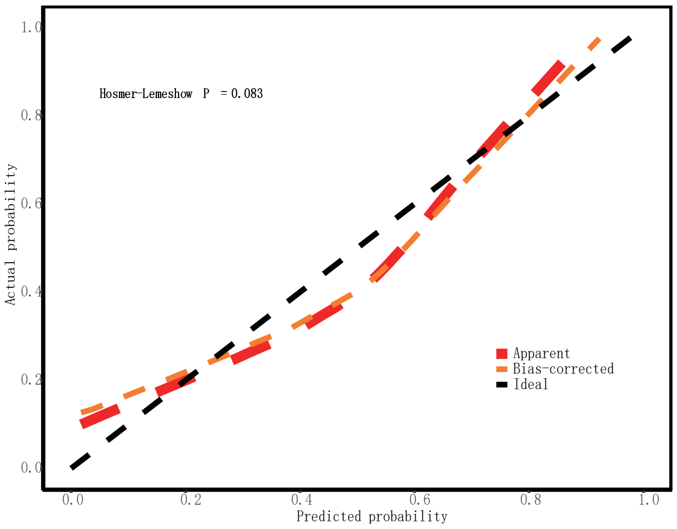

②HL test of Clinical-Radiomics modle

Derivation

```
Hosmer and Lemeshow goodness of fit (GOF) test

data:  fit_train$y, fitted(fit_train)
X-squared = 13.969, df = 8, p-value = 0.08258
```

Validation

```
Hosmer and Lemeshow goodness of fit (GOF) test

data:  fit_test$y, fitted(fit_test)
X-squared = 6.7995, df = 8, p-value = 0.5584
```

③DCA of Clinical-Radiomics modle

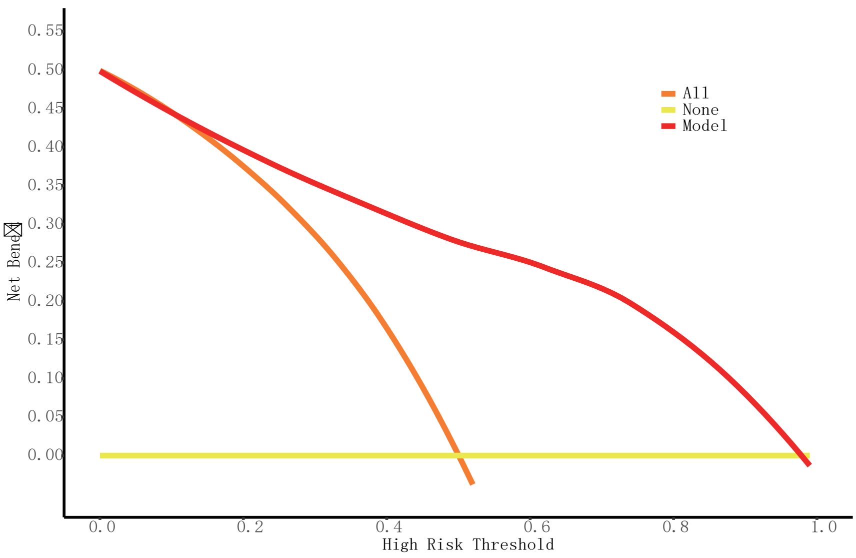

④Delong test of Clinical-Radiomics modle

```
DeLong's test for two ROC curves

data:  roc1 and roc2
D = 0.24221, df = 88.92, p-value = 0.8092
alternative hypothesis: true difference in AUC is not equal to 0
sample estimates:
AUC of roc1 AUC of roc2
  0.8677778   0.8528000
```

⑤Radiomic Score AUC

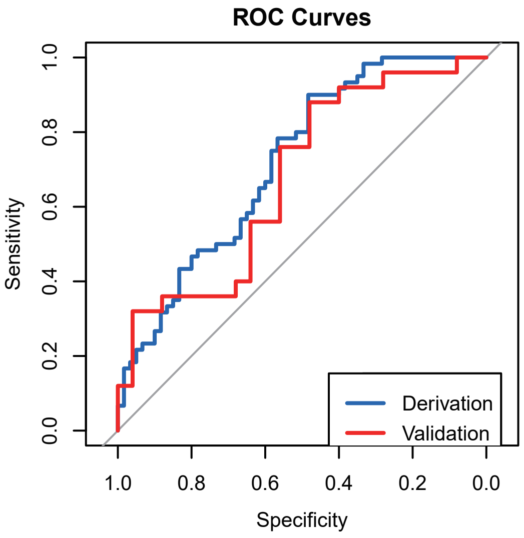

delong test

```
DeLong's test for two ROC curves

data:  roc1 and roc2
D = 0.46688, df = 85.867, p-value = 0.6418
alternative hypothesis: true difference in AUC is not equal to 0
sample estimates:
AUC of roc1 AUC of roc2
  0.7188889   0.6768000
```

Permutation test

```
Permutation Test
> cat("Permutation Test p-value:", perm_test_results$p_value, "\n")
Permutation Test p-value: 0.024
```
