# Supplementary material for: Development and validation of prediction models for special subtype of primary aldosteronism: patients with negative adrenal CT imaging
Source: Front Endocrinol (Lausanne). 2025 Jul 11;16:1563748. doi: 10.3389/fendo.2025.1563748 (PMC12289499; doi:10.3389/fendo.2025.1563748)
Supplement: Supplementary file 2 [file DataSheet2.pdf]

Supplementary material5: Supplementary statistical data

①Normal test

Shapiro-Wilk test

Sample	Statistic	*P value*
Age	0.994	0.895
Height	0.987	0.325
Weight	0.981	0.089
BMI	0.965	0.003
SBP	0.930	<.001
DBP	0.850	<.001
HTN Duration	0.818	<.001
Aldo_max	0.840	<.001
Renin	0.300	<.001
K^+(Min.)	0.976	0.027
Echo-IVSd	0.955	0.001
Echo-LVEF	0.810	<.001
RF-UA	0.991	0.603
RF-Cr	0.946	<.001
RF-eGFR	0.988	0.381
U-MA/Cr	0.450	<.001
U-K	0.915	<.001
24h U-K	0.865	<.001
BL-TC	0.985	0.216
BL-TG	0.661	<.001
BL-LDL	0.984	0.170
BL-HDL	0.935	<.001
24h AE(Max.)	0.890	<.001
Gender	0.624	<.001
Rad. Score	0.986	0.247
Clin-Rad. Score	0.976	0.029

②t-test AND Chi-square

Variables	Total (n = 120)	0 (n = 60)	1 (n = 60)	Statistic	*P*
Age, Mean ± SD	48.40 ± 9.66	48.95 ± 9.63	47.85 ± 9.75	t=0.62	0.535
Height, Mean ± SD	163.01 ± 7.76	163.18 ± 7.65	162.83 ± 7.92	t=0.25	0.806
Weight, Mean ± SD	67.96 ± 12.30	66.63 ± 11.36	69.28 ± 13.14	t=-1.18	0.239
RF-UA, Mean ± SD	339.33 ± 84.72	320.80 ± 85.55	358.17 ± 80.26	t=-2.46	**0.015**
RF-eGFR, Mean ± SD	96.02 ± 20.46	96.78 ± 20.14	95.26 ± 20.92	t=0.40	0.687
BL-TC, Mean ± SD	4.17 ± 0.86	4.07 ± 0.70	4.27 ± 0.98	t=-1.26	0.211
BL-LDL, Mean ± SD	2.46 ± 0.79	2.41 ± 0.68	2.50 ± 0.89	t=-0.59	0.559
Gender, n(%)				χ^2=2.79	0.095
1(male)	71 (59.17)	40 (66.67)	31 (51.67)		
2(female)	49 (40.83)	20 (33.33)	29 (48.33)		

t: t-test, χ^2: Chi-square test

SD: standard deviation

③ U test

	BMI	SBP	DBP	HTN Duration	Aldo_max	Renin
- U	1461.000	1570.500	1588.000	1668.000	1015.500	1317.500
W	3291.000	3400.500	3418.000	3498.000	2845.500	3147.500
Z	-1.779	-1.219	-1.128	-.695	-4.118	-2.535
	.075	.223	.259	.487	.000	.011

	K(Min.)	Echo-IVSd	Echo-LVEF	RF-Cr	U-MA/Cr	U-K
- U	1355.000	1063.500	1147.000	1753.000	1313.500	1407.500
W	3185.000	2288.500	2372.000	3523.000	2853.500	2838.500
Z	-2.338	-2.152	-1.588	-.090	-.043	-.616
	.019	.031	.112	.928	.966	.538

	24h U-K	BL-TG	BL-HDL	24h AE(Max.)	Rad. Score	Clin-Rad. Score
- U	1478.000	1098.000	1368.500	664.000	1012.000	476.000
W	2909.000	2868.000	3079.500	1610.000	2842.000	2306.000
Z	-.194	-3.342	-1.867	-3.712	-4.136	-6.949
	.846	.001	.062	.000	.000	.000

④ **Univariate Analysis + Multivariate Analysis**

Variables	Univariate					Multivariate				
	β	S.E	Z	*P*	OR (95%CI)	β	S.E	Z	*P*	OR (95%CI)
K^+(Min)	0.81	0.34	2.42	**0.015**	2.26 (1.17 ~ 4.37)	0.97	0.44	2.19	**0.028**	2.63 (1.11 ~ 6.26)
IVSd	-0.31	0.14	-2.29	**0.022**	0.73 (0.56 ~ 0.96)	-0.60	0.18	-3.25	**0.001**	0.55 (0.38 ~ 0.79)
UA	0.01	0.00	2.37	**0.018**	1.01 (1.01 ~ 1.01)	0.01	0.00	2.76	**0.006**	1.01 (1.01 ~ 1.02)
24h AE(Max)	-0.01	0.00	-3.73	**<.001**	0.99 (0.99 ~ 0.99)	-0.01	0.00	-2.76	**0.006**	0.99 (0.99 ~ 0.99)
Rad Score	1.84	0.46	4.01	**<.001**	6.30 (2.56 ~ 15.52)	1.92	0.57	3.36	**<.001**	6.85 (2.23 ~ 21.03)

OR: Odds Ratio, CI: Confidence Interval

⑤Confusion Matrix: Derivation Set and Validation Set (Performance before adjusting the cut-off value)

Data	AUC (95%CI)	Accuracy (95%CI)	Sensitivity (95%CI)	Specificity (95%CI)	PPV (95%CI)	NPV (95%CI)	cut off
Derivation	0.87 (0.80-0.93)	0.81 (0.73-0.87)	0.88 (0.80 - 0.96)	0.73 (0.62 - 0.85)	0.77 (0.67 - 0.87)	0.86 (0.77 - 0.96)	0.63
Temporal Validation	0.85 (0.75-0.96)	0.78 (0.64-0.88)	0.84 (0.70 - 0.98)	0.72 (0.54 - 0.90)	0.75 (0.59 - 0.91)	0.82 (0.66 - 0.98)	0.63
