# Supplementary material for: Development and validation of prediction models for special subtype of primary aldosteronism: patients with negative adrenal CT imaging
Source: Front Endocrinol (Lausanne). 2025 Jul 11;16:1563748. doi: 10.3389/fendo.2025.1563748 (PMC12289499; doi:10.3389/fendo.2025.1563748)
Supplement: Supplementary file 3 [file DataSheet3.pdf]

Supplementary materials for KNN model modeling process

Performance of KNN model

Metric	Train_Value	Test_Value	Train_CI 95%	Test_CI 95%
AUC	0.712	0.676	0.595-0.821	0.479-0.823
Sensitivity	0.564	0.667	0.413-0.721	0.472-0.871
Specificity	0.769	0.667	0.641-0.902	0.465-0.867
Accuracy	0.667	0.667	0.552-0.771	0.501-0.802
Error Rate	0.333	0.333		
F1	0.629	0.667		
Kappa	0.333	0.333		
best k value	41			

Confusion matrix of KNN model

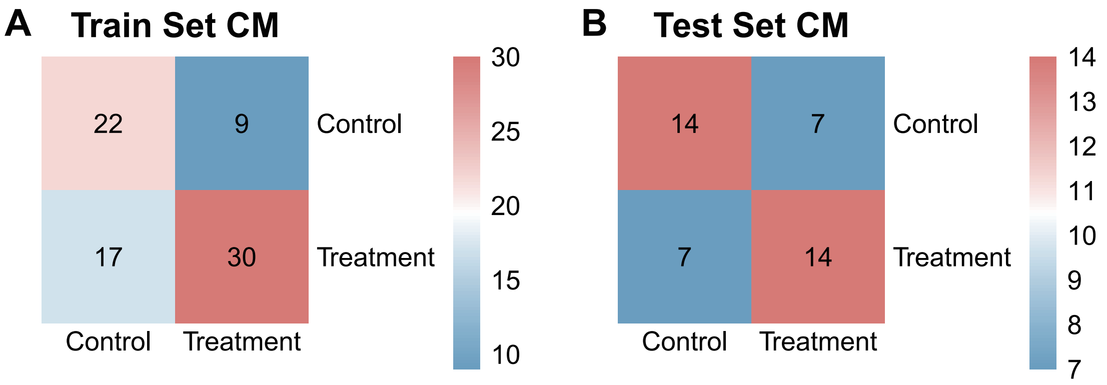

ROC curve of KNN model

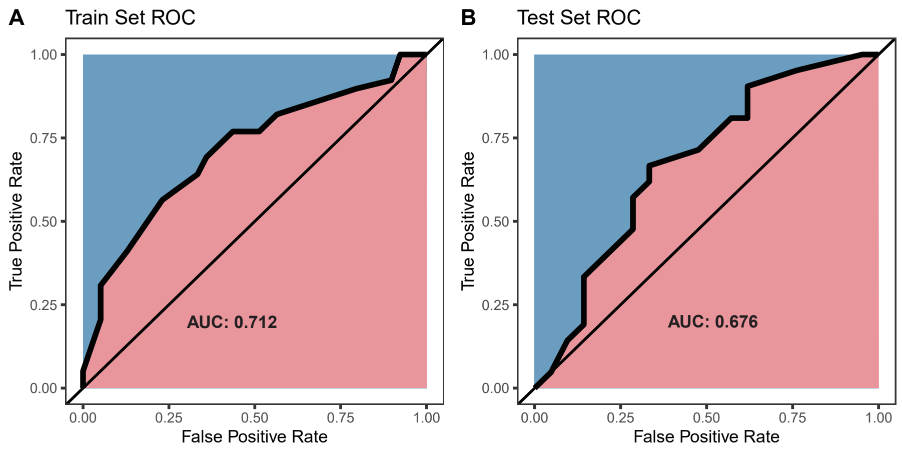

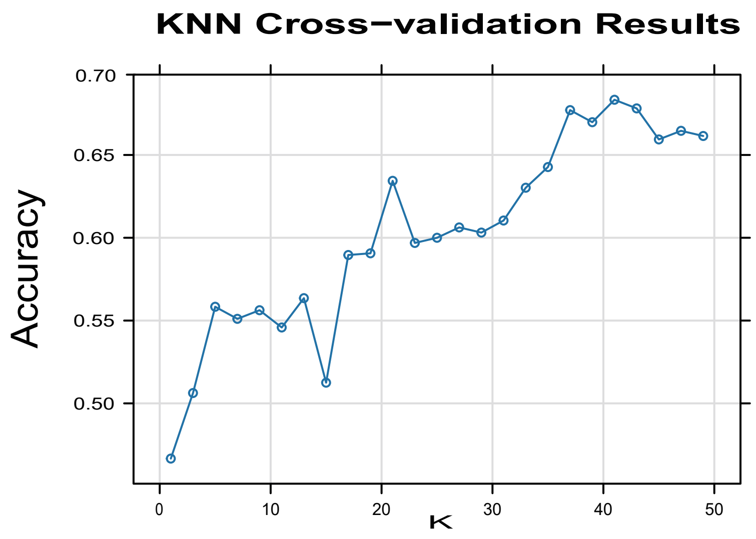

Multicollinearity test Pearson test thermodynamic chart results

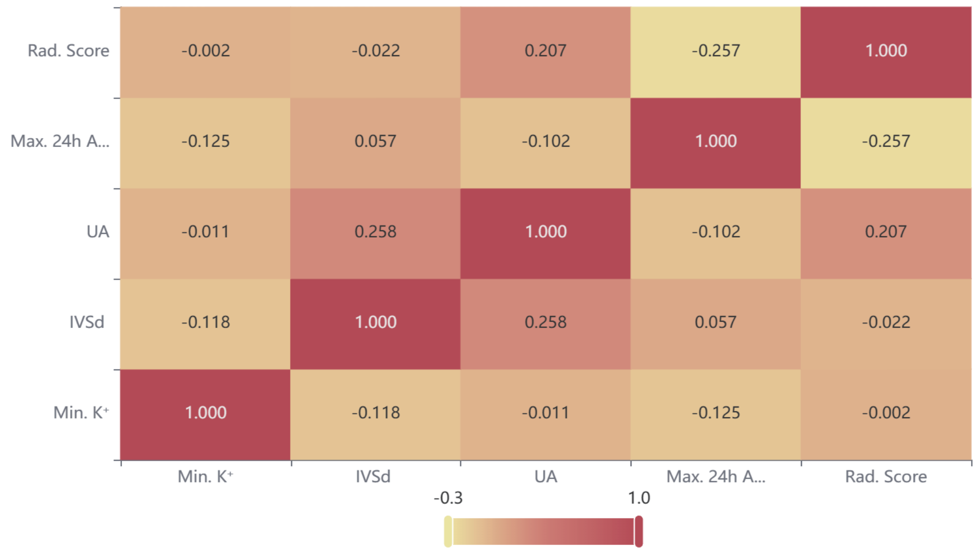

95% confidence interval of AUC

1.Lasso

> lasso_modelroc1

Call:

roc.default(response = SZtrain_label, predictor = lasso_Radscore_train, ci = T)

Data: lasso_Radscore_train in 39 controls (SZtrain_label 0) < 39 cases (SZtrain_label 1).

Area under the curve: 0.7179

95% CI: 0.6022-0.8337

> lasso_modelroc2

Call:

roc.default(response = SZtest_label, predictor = lasso_Radscore_test, ci = T)

Data: lasso_Radscore_test in 21 controls (SZtest_label 0) < 21 cases (SZtest_label 1).

Area under the curve: 0.6757

95% CI: 0.5074-0.8441

2.Ridge

> ridge_modelroc1

Call:

roc.default(response = SZtrain_label, predictor = ridge_Radscore_train, ci = T)

Data: ridge_Radscore_train in 39 controls (SZtrain_label 0) < 39 cases (SZtrain_label 1).

Area under the curve: 0.7344

95% CI: 0.6238-0.845

> ridge_modelroc2

Call:

roc.default(response = SZtest_label, predictor = ridge_Radscore_test, ci = T)

Data: ridge_Radscore_test in 21 controls (SZtest_label 0) < 21 cases (SZtest_label 1).

Area under the curve: 0.6939

95% CI: 0.5297-0.8581

3.LR

> modelroc_LR1

Call:

roc.default(response = LR_train$Label, predictor = LR_train_pred, levels = c("0", "1"), direction = "<", ci = T)

Data: LR_train_pred in 39 controls (LR_train$Label 0) < 39 cases (LR_train$Label 1).

Area under the curve: 0.7528

95% CI: 0.6459-0.8596

> modelroc_LR2 = roc(LR_test$Label,LR_test_pred,levels=c("0", "1"),direction="<",ci=T)

> modelroc_LR2

Call:
roc.default(response = LR_test$Label, predictor = LR_test_pred, levels = c("0", "1"), direction = "<", ci = T)
Data: LR_test_pred in 21 controls (LR_test$Label 0) < 21 cases (LR_test$Label 1).
Area under the curve: 0.6485
95% CI: 0.474-0.823

4.LDA

> roc_result = roc.test(modeLDAoc_LDA1,modeLDAoc_LDA2)
> modeLDAoc_LDA1
Call:
roc.default(response = lda_train$Label, predictor = LDA_train_pred, levels = c("0", "1"), direction = "<", ci = T)
Data: LDA_train_pred in 39 controls (lda_train$Label 0) < 39 cases (lda_train$Label 1).
Area under the curve: 0.7554
95% CI: 0.6494-0.8615
> modeLDAoc_LDA2
Call:
roc.default(response = lda_test$Label, predictor = LDA_test_pred, levels = c("0", "1"), direction = "<", ci = T)
Data: LDA_test_pred in 21 controls (lda_test$Label 0) < 21 cases (lda_test$Label 1).
Area under the curve: 0.6463
95% CI: 0.4737-0.8189

5.SVM

> modelroc_svm1
Call:
roc.default(response = SZtrain$Label, predictor = train_prob_svm[, 2], levels = c("0", "1"), direction = "<", ci = T)
Data: train_prob_svm[, 2] in 39 controls (SZtrain$Label 0) < 39 cases (SZtrain$Label 1).
Area under the curve: 0.7495
95% CI: 0.6404-0.8586
> modelroc_svm2
Call:
roc.default(response = SZtest$Label, predictor = test_prob_svm[, 2], levels = c("0", "1"), direction = "<", ci = T)
Data: test_prob_svm[, 2] in 21 controls (SZtest$Label 0) < 21 cases (SZtest$Label 1).
Area under the curve: 0.6372
95% CI: 0.4637-0.8107
